# Cat-Related Injuries and Rabies Exposure: A Three-Decade Retrospective Analysis from the Zagreb Antirabies Clinic (1995–2025)

**DOI:** 10.3390/v18080817

**Published:** 2026-07-25

**Authors:** Krešimir Tućin, Dora Primorac, Ivana Lojkić, Radovan Vodopija

**Affiliations:** 1 Institute of Public Health of Zagreb County, Mokrička ulica 54, 10290 Zaprešić, Croatia; 2 Andrija Štampar Teaching Institute of Public Health, Mirogojska ulica 16, 10000 Zagreb, Croatia; dora.primorac@stampar.hr (D.P.); radovan.vodopija@stampar.hr (R.V.); 3 Croatian Veterinary Institute, Savska cesta 143, 10000 Zagreb, Croatia; ilojkic@veinst.hr

**Keywords:** rabies, cat bite, cat scratch, animal bites, post-exposure prophylaxis, rabies immunoglobulin, lyssavirus, epidemiology, rabies elimination, Croatia

## Abstract

Cat-related injuries are a frequent reason for antirabies consultation, but long-term data on their epidemiology and management remain limited, particularly in European settings that have transitioned from endemic sylvatic rabies to officially rabies-free status. We retrospectively analysed registry data on all patients examined for cat-related injuries at the Zagreb Antirabies Clinic, the Croatian Reference Centre for Rabies, between 1995 and 2025. Among 24,107 animal-related consultations, 3147 (13.1%) involved cats. Most patients were female (64.8%), and children under 18 years of age accounted for 14.7% of the patients. The offending cat had a known owner in 48.5% of cases and was unknown in 45.9%. Records associated with laboratory-confirmed rabid cats accounted for 4.8%, all from 1995 to 2012. Post-exposure prophylaxis (PEP) was administered to 37.6% of patients, and human rabies immunoglobulin (HRIG) to 2.9%. Hospitalisation was rare (1.1%), while injuries most often involved the fist and fingers (59.5%). PEP administration was strongly associated with the rabies risk status of the offending cat, increasing from 8.7% for known-owner cats to 62.0% for unknown cats and approximately 87% for suspected or laboratory-confirmed rabid cats (*p* < 0.001). In multivariable analysis, animal status remained the principal factor independently associated with PEP (adjusted odds ratio for unknown versus known-owner cats approximately 18), whereas patient characteristics had limited effect. HRIG use declined below 1% in 2015–2025, paralleling the elimination of sylvatic rabies in Croatia. Continued structured exposure assessment remains relevant despite rabies-free status, particularly when cats are unknown or unavailable for observation.

## 1. Introduction

Rabies is an acute progressive encephalomyelitis caused by negative-strand RNA viruses of the genus *Lyssavirus*, family *Rhabdoviridae*, order *Mononegavirales*. Although it is almost invariably fatal once clinical signs appear, it is also entirely vaccine-preventable; nevertheless, it is still responsible for an estimated 59,000 human deaths annually, predominantly in Asia and sub-Saharan Africa, where dog-mediated transmission prevails [1,2,3,4]. In recognition of this preventable burden, the World Health Organization (WHO), the World Organisation for Animal Health (WOAH) and the Food and Agriculture Organization (FAO) have set the goal of eliminating human deaths from dog-mediated rabies by 2030 [5,6]. The cornerstone of human rabies prevention remains timely post-exposure prophylaxis (PEP), comprising immediate wound care, rabies vaccination and, for severe (category III) exposures, the co-administration of human rabies immunoglobulin (HRIG) [5].

In Europe, human rabies has become exceedingly rare, largely as a result of sustained oral rabies vaccination (ORV) campaigns directed at wildlife reservoirs. In the Republic of Croatia, the last indigenous human case was recorded in 1964, with only two subsequent imported cases, in 1989 and 1996 [7]. Sylvatic rabies, maintained mainly by the red fox (*Vulpes vulpes*), persisted for decades until ORV campaigns, launched in 2011 and conducted twice yearly, achieved a steady decline; oral vaccination of foxes has been the decisive tool in eliminating fox-mediated rabies across large parts of Western and Central Europe over the past three decades [8,9,10], and the European Union has long identified rabies elimination as a priority [11]. The last animal rabies case in Croatia was recorded in 2014, and in 2021 the European Commission officially declared Croatia free of rabies [7,12,13]. Although Croatia has been officially rabies-free since 2021, antirabies-clinic (ARC) consultations for cat-related injuries have continued at a mean of approximately 73 per year in the most recent, rabies-free period (2021–2025), and PEP has still been administered to roughly one in three of these patients, supporting the continued maintenance of antirabies services and structured exposure assessment [12,14,15].

Among the animals responsible for human injuries, dogs have traditionally received the greatest attention, both because of their role in dog-mediated rabies and because they account for the majority of bite injuries worldwide. Cats, however, represent an important and often second-ranking source of mammalian bites and scratches in several international settings, including Europe, yet their epidemiology has been comparatively neglected [16,17]. The slender, sharp dentition of cats produces deep, narrow puncture wounds that inoculate bacteria into poorly vascularised deep tissues, resulting in markedly higher rates of wound infection than dog bites, with pathogens such as *Pasteurella multocida*, *Bartonella henselae* (the agent of cat-scratch disease) and *Capnocytophaga* spp. being of particular concern [18,19,20,21]. Cats are also recognised as susceptible to lyssaviruses. Although they are not viral reservoirs, they may act as incidental hosts and, in specific circumstances, as bridge hosts for lyssavirus transmission to humans, becoming infected with whichever lyssavirus predominates in their geographic locality, with documented spill-over events involving the European bat lyssavirus type 1 (EBLV-1) in France and the West Caucasian bat virus (WCBV) in Italy [15,22,23,24]. Bats are the principal reservoirs of lyssaviruses in Europe, and bat rabies surveillance remains an integral component of rabies monitoring on the continent [25,26].

From a public health perspective, every cat-related injury presenting to an antirabies clinic requires individualised rabies risk assessment, in which the vaccination and observation status of the offending animal is decisive. When the animal is owned, healthy and available for veterinary observation, PEP can frequently be withheld or discontinued; when the animal is unknown, unavailable, or behaving abnormally, PEP is indicated according to WHO recommendations [5]. Globally, the public health relevance of feline rabies is increasingly emphasised in recent reviews: in the United States, cats consistently account for more positive rabies cases per year than dogs (approximately 1% of submitted feline samples test positive, compared with 0.3% of canine samples), and in Europe roughly one third of currently reported rabies virus (RABV) cases in domestic animals are now found in cats, reflecting the success of canine rabies control and the increasing relative importance of feline exposures [15]. In parts of the Americas the concern is more acute still: a recent three-decade analysis from Colombia documented a shift from dog- to cat-transmitted human rabies, with unvaccinated free-roaming cats bridging the bat-maintained virus to mostly young, rural patients [27]. Such settings, where a wildlife reservoir persists, differ from rabies-free countries, where cat injuries raise a question of exposure assessment and prophylaxis rather than of ongoing transmission. How these decisions are applied in practice, and how they have changed across both the endemic and the rabies-free periods, has rarely been described from a European reference centre.

Fehlner-Gardiner et al. recently consolidated the global picture of rabies in cats as an emerging public health concern across five continents [15], but their review explicitly notes a relative paucity of long-term, individual-level data from European reference centres during the transition from sylvatic endemicity to officially rabies-free status. We addressed this gap with three uninterrupted decades of clinic-level data from the Croatian Reference Centre for Rabies, hypothesising that PEP is administered to a substantial proportion of patients with cat-related injuries, and that this proportion is strongly associated with the status of the offending animal, being highest when the animal is unknown or assessed as high-risk. The specific aims were: (I) to describe the demographic and clinical characteristics of injured persons; (II) to characterise injury localisation and animal status; (III) to describe PEP and HRIG administration according to animal status and time period; and (IV) to examine temporal trends across a period that encompasses the elimination of rabies in Croatia.

## 2. Materials and Methods

### 2.1. Study Design and Setting

This was a retrospective, registry-based observational study of all patients examined for cat-related injuries at the Zagreb Antirabies Clinic, the Croatian Reference Centre for Rabies, operating within the Andrija Štampar Teaching Institute of Public Health in Zagreb, Croatia, between 1 January 1995 and 31 December 2025. The clinic serves the city of Zagreb and the surrounding region and represents the national reference point for rabies exposure assessment and PEP. The study period was selected to capture three full decades of continuous registry data, spanning both the endemic sylvatic-rabies era and the period following the declaration of Croatia as a rabies-free country in 2021. Because the analysis is based on persons who actively presented to the ARC, the denominators throughout reflect clinic-based consultations rather than population exposures; the true population incidence of cat-related injuries in the catchment area cannot be estimated from these data, as the number of exposed persons who did not seek medical care is unknown.

### 2.2. Data Source, Study Population and Case Definition

Data were extracted from the official patient registry of the Zagreb ARC for the entire study period. For the purposes of this study, a cat-related injury was defined as a documented physical contact with a cat (bite, scratch, or saliva contamination of a wound or mucous membrane) that prompted ARC consultation and was considered by the attending clinician to constitute a potential rabies exposure. All patients meeting this definition were eligible for inclusion; no exclusion criteria were applied, and the analysis therefore encompasses the complete population of cat-related presentations over the 31-year period. The registry also captures persons registered solely as epidemiological contacts of a confirmed or suspected rabid animal in the absence of any physical exposure (for example, household members who had stroked a confirmed rabid cat without sustaining bite, scratch or mucosal contamination); such persons were retained in the dataset for completeness but were not eligible for PEP under WHO criteria and are flagged where relevant in the results. The registry does not distinguish bite from scratch as separate fields, and the two cannot be disaggregated for this analysis. For context, the total number of patients examined at the clinic for any animal-related injury during the same period was retrieved from the annual aggregate statistics of the clinic and used to express cat-related injuries as a proportion of the overall antirabies workload.

### 2.3. Variables and Definitions

The following variables were retrieved for each patient: year of the incident; patient age and sex; status of the offending animal; anatomical localisation of the injury; administration of PEP; vaccination schedule; vaccine type; number of vaccine doses received; administration of HRIG; course of treatment (continuation, non-attendance for scheduled doses, or discontinuation); veterinary observation of the animal; laboratory testing of the animal for rabies; and hospitalisation. The status of the offending animal was classified according to the Croatian ABCD categorisation, a national scheme defined in Croatian public health regulations [28] and used in routine antirabies practice at the Croatian Reference Centre for Rabies [29]: category A, an injury caused by a laboratory-confirmed rabid cat (or other contact with such an animal or contaminated material); category B, an injury caused by a cat clinically suspected of rabies; category C, an injury caused by a cat that is unknown, dead, stray, killed, or feral, and therefore unavailable for observation; and category D, an injury caused by a cat with a known owner that remained healthy after a 10-day period of veterinary observation. The ABCD scheme thus describes the status and availability of the offending animal, whereas the WHO category I/II/III system describes the nature of the human–animal exposure; the WHO category was applied at the clinical level but was not extracted as a separate variable from the registry. The same ABCD scheme has recently been applied in another Croatian antirabies cohort, from Split-Dalmatia County [30]. Because the registry is patient-based, animal-status categories refer to patient records and consultations rather than necessarily to unique animals. Therefore, we analysed animal status as recorded in the registry, because this was the most consistently available variable for rabies risk assessment during the entire period of the study. Injury localisation was grouped as fist and fingers, upper limbs, lower limbs, head and neck, trunk, or multiple sites. PEP was defined as the administration of at least one dose of rabies vaccine following the exposure. The Zagreb (2-1-1) intramuscular regimen and the Essen (1-1-1-1-1) regimen were recorded as the principal vaccination schedules, in accordance with WHO-endorsed protocols [5,31]. A course was considered complete when a full schedule was administered at the ARC: four doses under the Zagreb 2-1-1 regimen, or an Essen course of four, five or six doses (the more recent four-dose schedule, the historical five-dose schedule, or an extended six-dose course given for high-risk exposures). Because the registry captures only doses administered at the Zagreb ARC, a dose count below a full course does not necessarily indicate incomplete prophylaxis. Such patients included those referred for continuation of a course begun at another institution or abroad, those who did not return for scheduled doses, and those who discontinued treatment. Recorded dose counts were therefore interpreted as doses administered at this clinic rather than as a measure of overall PEP completeness. WHO-recommended regimens, vaccine types and immunoglobulin use are detailed in the current position paper and expert consultation [5,31,32], and standardised laboratory techniques underpin the confirmation of animal rabies [33]. The registry also contained a binary code corresponding to a clinical impression of cat-scratch disease (CSD); however, because the consistency of this coding across the 31-year study period could not be independently validated, the CSD variable was excluded from all analyses presented here.

### 2.4. Statistical Analysis

Categorical variables were summarised as absolute and relative frequencies, and continuous variables (age) as medians with interquartile ranges (IQRs), given their non-normal distribution. Associations between categorical variables were assessed using Pearson’s χ^2^ test, or Fisher’s exact test where expected cell counts were small; Cramér’s V was used as a measure of effect size for the larger contingency tables. Differences in age distribution between groups were evaluated using the Mann–Whitney U test for two groups and the Kruskal–Wallis test for more than two groups, given the non-normal distribution of age. Temporal trends were examined by grouping the data into three decadal periods (1995–2004, 2005–2014 and 2015–2025) and, for continuous year-on-year trends, by Spearman’s rank correlation. The decadal grouping was descriptive; formal testing of the temporal trend used the segmented (interrupted time-series) regression detailed below. A two-tailed *p*-value below 0.05 was considered statistically significant. Given the large sample size, which renders even modest differences statistically significant, interpretation emphasised the magnitude of associations (effect sizes such as Cramér’s V) rather than statistical significance alone. In addition, to identify factors independently associated with PEP administration, a multivariable binomial logistic regression model was fitted, with animal status (reference category: known owner), sex, age, injury localisation and study period entered as covariates; adjusted odds ratios (aORs) with 95% confidence intervals (CIs) are reported; model discrimination was summarised by the area under the ROC curve and multicollinearity by variance inflation factors. To assess how the temporal trend in PEP administration changed in relation to the key events of the national rabies programme, a segmented (interrupted time-series) binomial logistic regression was fitted, with calendar year as the underlying time variable and the last recorded indigenous rabies case (2014) as the interruption point; the model estimated the pre-2014 trend, any abrupt change in level at 2014, and the change in slope thereafter. Additional interruption points at the introduction of oral fox vaccination (2011) and at the formal rabies-free declaration (2021) were also examined. These analyses were performed using the standard binomial logistic regression module with the time, level-change and slope-change variables constructed as covariates. Data were compiled and managed in Excel (Microsoft 365; Microsoft Corporation, Redmond, WA, USA), and statistical analyses were performed using jamovi (version 2.7.30; The jamovi Project, Sydney, NSW, Australia).

### 2.5. Ethical Considerations

The study was conducted in accordance with the principles of the Declaration of Helsinki and was approved by the Ethics Committee of the Andrija Štampar Teaching Institute of Public Health (protocol code 03/2026; reference numbers KLASA 641-01/26-01/1, URBROJ 251-758-26-9; date of approval 5 May 2026). Because the study was based on the retrospective analysis of fully anonymised registry data, with no direct contact with the subjects, the requirement for individual informed consent was waived by the Ethics Committee. All personal data were processed in anonymised form, each subject being identified solely by a numerical code without any possibility of direct identification, and access to the source records was restricted to authorised investigators, in compliance with applicable data-protection regulations.

## 3. Results

### 3.1. Overall Workload and Patient Characteristics

**Figure 1:**
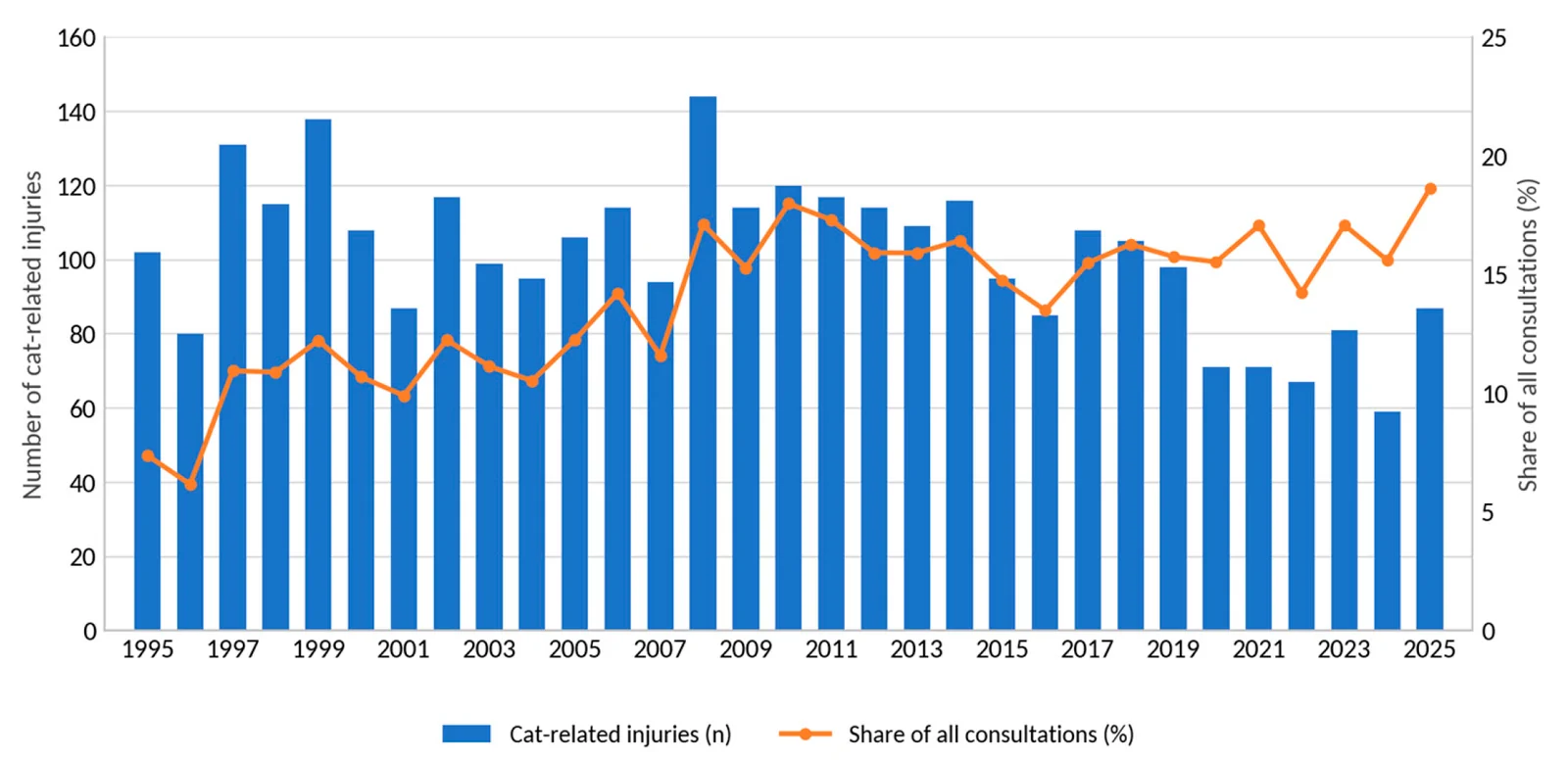
Annual number of cat-related injuries (bars) and their share (%) of all rabies-clinic consultations (line), Zagreb Antirabies Clinic, 1995–2025.

Between 1995 and 2025, a total of 24,107 patients were examined at the Zagreb ARC for injuries caused by animals, of whom 3147 (13.1%) had sustained cat-related injuries. Cat-related injuries thus represented a substantial and consistent component of the workload of the clinic, with an average of 101.5 patients per year, ranging from 59 in 2024 to 144 in 2008 (Figure 1). Although the absolute number of cat-related consultations declined across the study period, the share of cat-related injuries among all antirabies consultations rose from a mean of 10.2% in 1995–2004 to 15.8% in 2015–2025 (Spearman’s ρ = 0.76, *p* < 0.001), because the overall antirabies workload contracted more steeply than cat-related presentations. This divergence indicates that cat exposures became relatively more prominent even as their absolute frequency fell. This partly reflects a contracting overall consultation denominator rather than a change in feline exposure risk alone, and is relevant to the interpretation of the temporal trends.

Women predominated, accounting for 2039 patients (64.8%) compared with 1108 men (35.2%). The median age was 44 years (IQR 25–59; range 1–93). Children under the age of 18 accounted for 462 patients (14.7%). Female patients were significantly older than male patients (median 46 versus 38 years; Mann–Whitney U test, *p* < 0.001). The full demographic and clinical profile of the study population is presented in Table 1.

### 3.2. Animal Status and Injury Localisation

In almost half of the cases the injury was caused by a cat with a known owner (category D; 1525 patients; 48.5%), while unknown animals (category C) accounted for a further 1444 patients (45.9%). Animals clinically suspected of rabies (category B) were involved in 28 patients (0.9%), and laboratory-confirmed rabid cats (category A) in 150 patients (4.8%). Laboratory testing results were recorded in 258 exposure records, of which 150 were positive and 108 negative for rabies (performed at the Croatian Veterinary Institute, Zagreb, the National Reference Laboratory for Rabies).

Injuries to the fist and fingers were by far the most frequent, occurring in 1872 patients (59.5%), followed by the upper limbs (579; 18.4%), lower limbs (419; 13.3%), multiple sites (202; 6.4%), head and neck (64; 2.0%) and trunk (11; 0.3%). The distribution of injury localisation differed significantly by sex (χ^2^ = 40.60, *df* = 5, *p* < 0.001): men were more frequently injured on the fist and fingers (65.7% versus 56.1%), whereas women more often sustained injuries to the lower limbs (15.3% versus 9.7%).

### 3.3. Post-Exposure Prophylaxis and Immunoglobulin

Rabies PEP was administered to 1183 patients (37.6%). Of these, 1091 received the rabies vaccine alone, while 92 (2.9% of the entire cohort) received the vaccine together with HRIG; all HRIG recipients also received the vaccine, as expected in HRIG-containing PEP regimens [5]. Among PEP recipients, the Zagreb regimen was by far the most frequently used schedule (1092 patients; 92.3%), followed by the Essen regimen (77; 6.5%). The most commonly administered vaccine was the purified chick embryo cell vaccine (752 patients, 63.6%), followed by human diploid cell vaccines. A complete course administered at the ARC (four doses under the Zagreb regimen or four to six doses under the Essen regimen) was documented in 827 PEP recipients (69.9%).

The administration of PEP was strongly associated with the status of the offending animal (χ^2^ = 1098, *df* = 3, *p* < 0.001; Cramér’s V ≈ 0.59). PEP was given to only 132 of 1525 patients (8.7%) injured by cats with a known owner, but to 896 of 1444 patients (62.0%) injured by unknown cats, 25 of 28 patients (89.3%) injured by suspected animals and 130 of 150 patients (86.7%) injured by confirmed rabid cats. These status-specific proportions are calculated over all registry records in each category, including a small number of non-exposure contacts, not only clinically exposed persons. A parallel gradient was observed for HRIG, which was administered to 44.7% of patients exposed to confirmed rabid cats and 42.9% of those exposed to suspected animals, but to fewer than 1% of those exposed to owned or unknown cats (Fisher’s exact test, *p* < 0.001). Veterinary observation of the animals was almost exclusively undertaken for owned animals (436/1525; 28.6%) and, to a lesser extent, for unknown cats (70/1444; 4.8%), and was never recorded for suspected or confirmed rabid animals, reflecting their clinical or laboratory disposition (Table 2).

Twenty of the 150 patients registered in association with a laboratory-confirmed rabid cat did not receive PEP. Inspection of the source records showed that these were registered as epidemiological contacts of a confirmed rabid animal (typically family members or neighbours linked by reference number to an index case) who had had no documented bite, scratch or mucosal exposure (for example, persons who had only stroked the animal on its back). They did not meet WHO criteria for any exposure category requiring PEP, and rabies prophylaxis was therefore correctly withheld. These contact-only registrations are retained in Table 1 only for registry completeness and for sensitivity and interpretive purposes, and represent the denominator of a registry rather than clinically exposed individuals.

**Figure 2:**
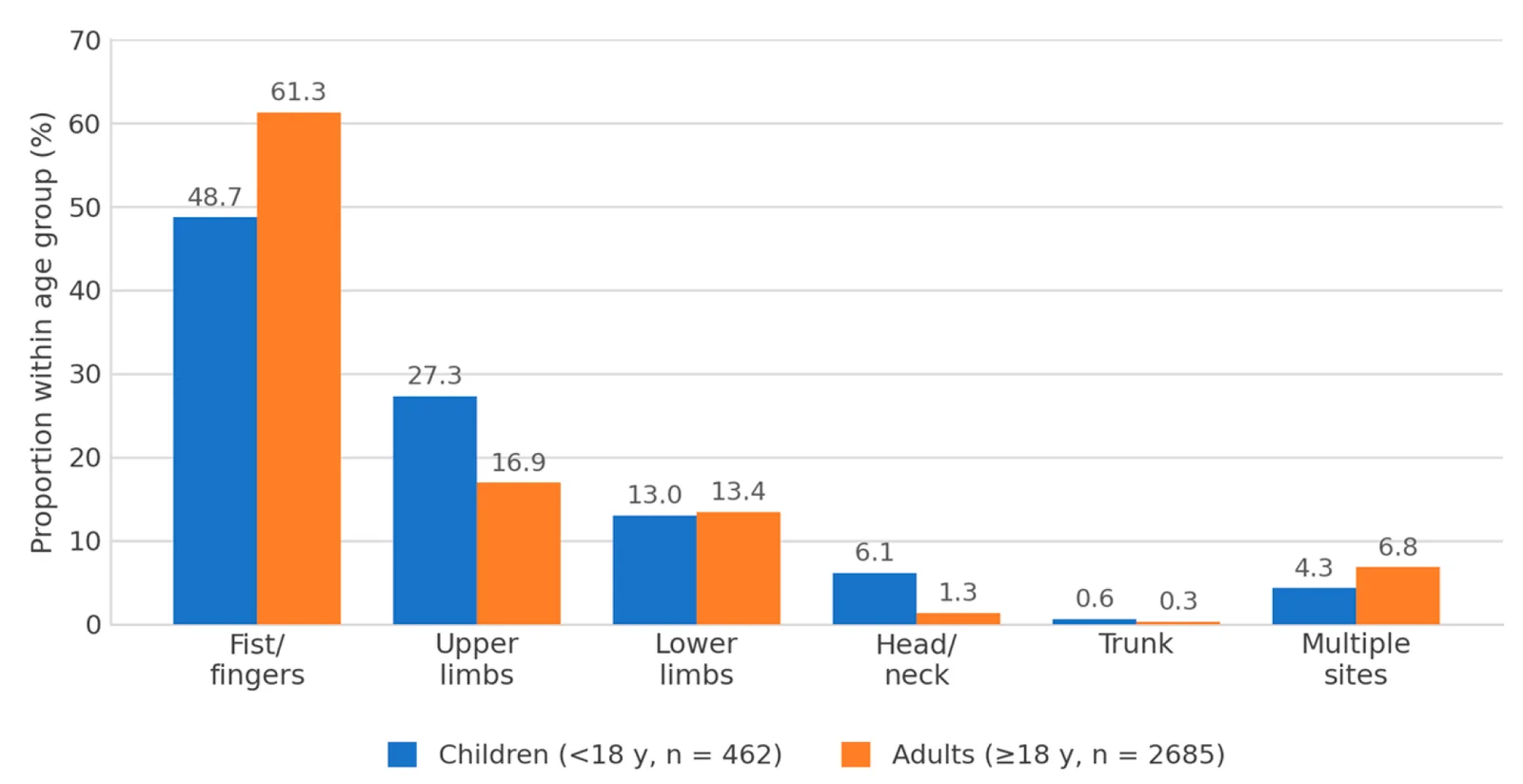
Distribution of injury localisation in children (<18 years, *n* = 462) versus adults (≥18 years, *n* = 2685). χ^2^ for the full six-category table = 82.06, *df* = 5, *p* < 0.001.

Two further patterns of clinical significance emerged when stratified by sex and age. Men received PEP more frequently than women (42.4% versus 35.0%; χ^2^ = 16.67, *df* = 1, *p* < 0.001). The crude proportion of PEP did not differ between children and adults (35.9% versus 37.9%; χ^2^ = 0.56, *df* = 1, *p* = 0.46). However, children were significantly more likely than adults to sustain injuries to the head and neck (6.1% versus 1.3%; χ^2^ for the full six-category localisation table = 82.06, *df* = 5, *p* < 0.001; Figure 2).

### 3.4. Clinical Outcomes and Course of Treatment

Hospitalisation was rare, recorded in only 36 patients (1.1%); the indication for admission was not consistently recorded and could relate to wound management, clinical observation, or comorbidity rather than to rabies risk as such. Among the 1183 patients who commenced PEP, 827 (69.9%) received a complete course at the ARC: 774 under the four-dose Zagreb regimen and 53 under the Essen regimen (five doses in 44, six doses in six, four doses in three). The remaining 356 (30.1%) had a non-standard dose count recorded at this clinic; in 232 the registry documented continuation of a course begun elsewhere, non-attendance for scheduled doses, or discontinuation, while in 124 no reason was recorded. As the registry captures only doses given at this clinic, these counts should not be equated with overall PEP completeness. With respect to protocol adherence, PEP initiation was concordant with the recommended risk-based indication: prophylaxis was withheld in 91.3% of exposures to owned, observable cats and administered in 62.0% of unknown-cat exposures and in approximately 87% of suspected or confirmed exposures, while the 20 contact-only registrations linked to a confirmed rabid cat correctly received no PEP (Section 3.3). Adherence to schedule completion could be assessed only for doses given at this clinic. Individual dose dates are recorded in the registry but were not extracted for the present analysis, so dose timeliness (day-0 initiation and inter-dose intervals) was not evaluated.

### 3.5. Temporal Trends, 1995–2025

**Figure 3:**
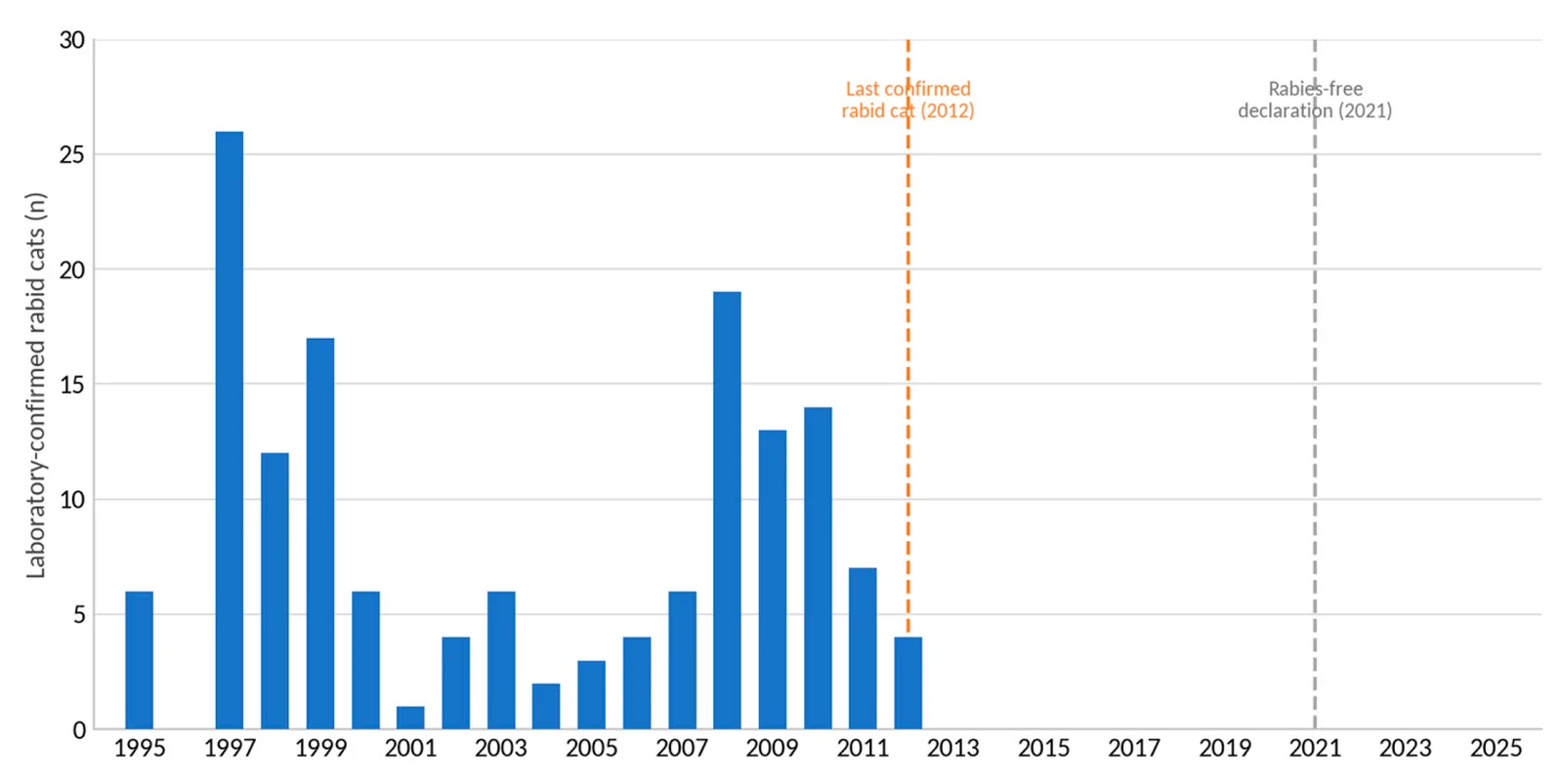
Annual number of patient records associated with laboratory-confirmed rabid cats, 1995–2025. All 150 confirmed cases occurred between 1995 and 2012. Vertical lines mark the year of the last confirmed rabid cat in the series (2012) and the European Commission declaration of Croatia as a rabies-free country (2021).

The annual number of cat-related injuries showed a significant overall decline across the three decades (Spearman’s ρ = −0.48, *p* = 0.006), although relative share of cat-related antirabies consultations did not show a comparable decline and became more prominent in later years (Figure 1). The clearest temporal pattern concerned laboratory-confirmed rabid cats: all 150 such patient records occurred between 1995 and 2012, peaking in 1997 (*n* = 26), with no confirmed rabid cat recorded from 2013 onwards (Figure 3). This pattern coincides closely in time with the documented decline of sylvatic rabies in Croatia: the last confirmed rabid cat in our series was recorded in 2012, two years before the last confirmed animal rabies case nationally (2014), and nine years before the European Commission declaration of rabies-free status in 2021.

**Figure 4:**
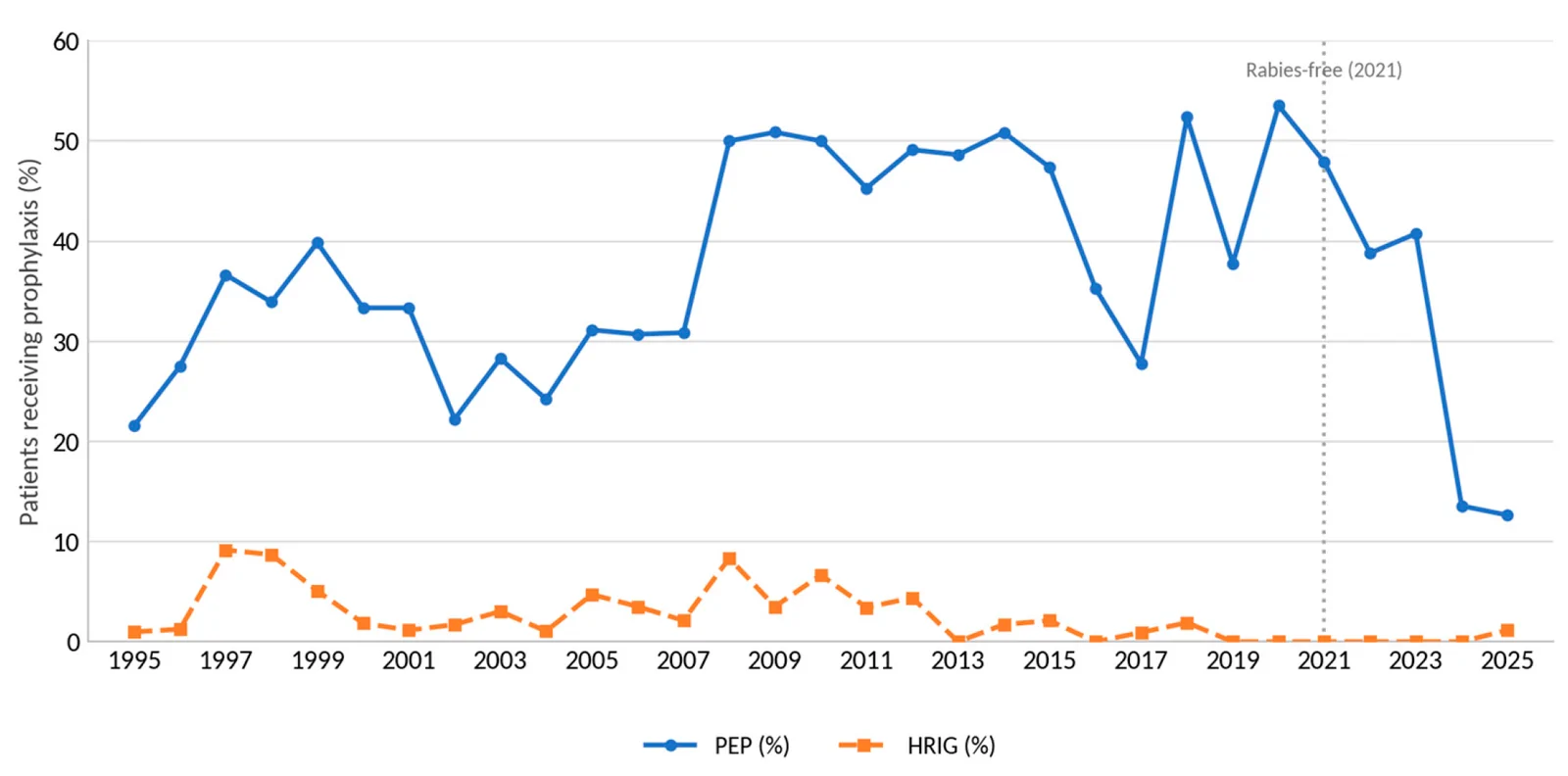
Annual proportions of patients receiving rabies post-exposure prophylaxis (PEP, solid line) and human rabies immunoglobulin (HRIG, dashed line), 1995–2025. The vertical dotted line marks the European Commission declaration of Croatia as a rabies-free country in 2021.

**Figure 5:**
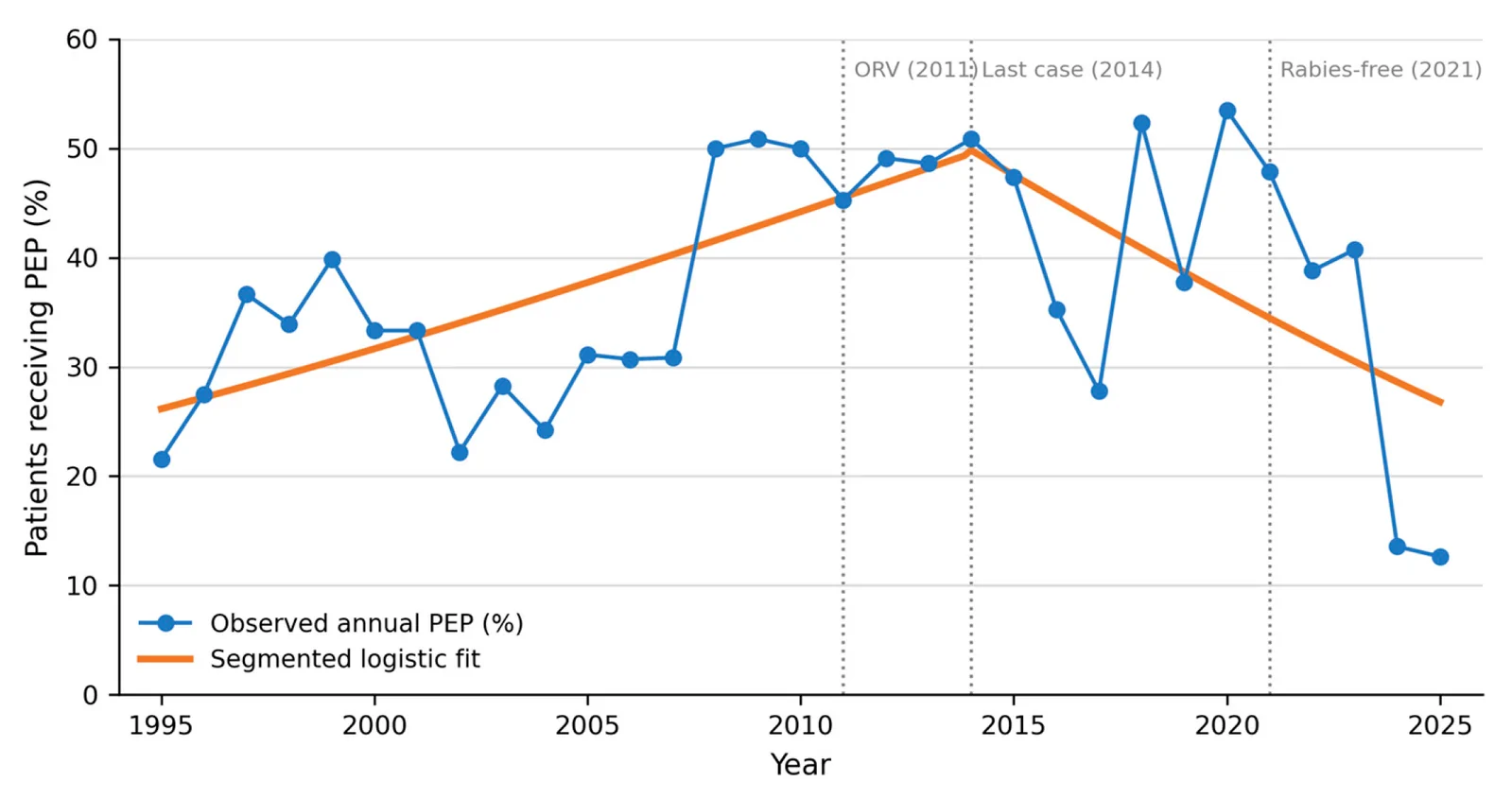
Segmented (interrupted time-series) analysis of the proportion of patients receiving post-exposure prophylaxis (PEP), 1995–2025. Points are observed annual proportions; the solid line is the fitted segmented logistic regression with an interruption at the last indigenous rabies case (2014). In the fitted model, the odds of receiving PEP increased up to the pre-specified 2014 interruption point and declined thereafter. Dotted vertical lines mark the introduction of oral fox vaccination (2011), the last indigenous case (2014), and the European Commission declaration of Croatia as a rabies-free country (2021).

The proportion of patients receiving PEP differed significantly across the three periods (χ^2^ = 44.1, *df* = 2, *p* < 0.001), rising from 30.6% in 1995–2004 to 44.3% in 2005–2014, before settling at 37.4% in 2015–2025 (Figure 4). The administration of HRIG, by contrast, declined progressively, from 3.7% and 4.0% in the first two periods to only 0.6% in 2015–2025, consistent with the disappearance of confirmed rabid animals. PEP continued to be administered to roughly one-third of patients even after the rabies-free declaration, reflecting the persistence of unknown-animal exposures and continued precautionary management (Figure 4). Notably, this proportion fell markedly in the final two years (to 13.6% in 2024 and 12.6% in 2025); this period is too short to establish a trend, but the decline may signal change in exposure-management practice after the rabies-free country status. The temporal trend in PEP administration was non-monotonic, rising over the earlier years to a plateau in the late 2000s and declining thereafter. In a segmented (interrupted time-series) model with the interruption pre-specified at the last indigenous rabies case (2014), the odds of receiving PEP had been rising beforehand (slope OR per year 1.055, 95% CI 1.038–1.073, *p* < 0.001) and fell afterwards (slope-change OR 0.866, 95% CI 0.831–0.901, *p* < 0.001; net post-2014 OR per year 0.913, 95% CI 0.880–0.948), with no abrupt change in level at the event itself (OR 1.01, 95% CI 0.76–1.34, *p* = 0.94); this reversal remained significant when the two most recent years were excluded (slope-change OR 0.94, *p* = 0.01) (Figure 5). Testing the other programme milestones, the introduction of oral fox vaccination in 2011 and the formal rabies-free declaration in 2021, likewise showed a gradual change in slope rather than an abrupt step, and the turning point in the observed trend preceded these milestones; the apparent change after 2021 depended solely on the sharp fall in the final two years and did not persist when those years were excluded. The decline in PEP administration therefore appears to reflect the gradual elimination of sylvatic rabies rather than an abrupt response to any single programme event.

Temporal trends were also evident in the distribution of animal status: the share of injuries attributable to unknown animals increased over time (from 38.6% in 1995–2004 to 51.9% in 2015–2025), while confirmed rabid animals disappeared entirely after 2012 (Table 3).

### 3.6. Factors Independently Associated with PEP

In the multivariable logistic regression model, animal status was the factor most strongly and independently associated with PEP: relative to known-owner cats, the adjusted odds of PEP were 17.9-fold higher for unknown cats and substantially higher again for suspected and laboratory-confirmed rabid cats, whereas sex, age and injury localisation had limited independent effect. The higher odds of PEP in the two later periods persisted after adjustment for animal status (Table 4). The model discriminated well (area under the ROC curve 0.85), and multicollinearity was negligible (all variance inflation factors below 1.4).

## 4. Discussion

In this 31-year retrospective analysis from the Zagreb Antirabies Clinic, the Croatian Reference Centre for Rabies, cat-related injuries accounted for 13.1% of all animal-related consultations, with PEP administered to 37.6% of patients and HRIG to 2.9%. The clearest factor associated with PEP use was the status of the offending cat: PEP rates rose from 8.7% for known-owner cats to 62.0% for unknown cats and approximately 87% for suspected or laboratory-confirmed rabid cats. Other statistically significant associations (of sex and age with injury localisation, and of time period with PEP, HRIG, and animal status) were of small magnitude (Cramér’s V 0.09–0.16). Our two prior expectations were met: PEP was given to more than one-third of patients, and its use was strongly associated with the status of the offending cat. Because animal status directly informs the post-exposure decision, however, this association largely reflects protocol-consistent practice rather than an independent predictor. In multivariable analysis this pattern persisted: after adjustment for sex, age, injury site and period, animal status remained by far the strongest correlate of PEP (unknown vs. owned cat, aOR ≈ 18), whereas patient characteristics contributed comparatively little (Table 4). To our knowledge, this is one of the larger single-centre series of cat-related injuries in Europe, and one of the few spanning both the endemic and the rabies-free era of a previously affected country [7,12,13], adding a European perspective to the growing evidence on rabies in cats as a public health issue [15].

The demographic profile of our cohort (female predominance 64.8%, median age in the mid-forties, and 14.7% children) is broadly consistent with the international literature, where a clear female excess has been reported in settings as diverse as Uruguay (66.5%) [16], Spain and Dallas (53–59%) [34,35], a large emergency-department series in California [36], and the Swiss sentinel network [17]. This may reflect differential exposure, as women are more likely to keep and handle cats [34,35]. The predominance of fist and finger injuries (59.5%) likewise reproduces a consistent pattern, with the upper limbs preferentially affected by cats in contrast to the lower-limb predominance of dog bites [17,34,36,37,38]. Fist and finger injuries are clinically relevant because the slender feline dentition produces deep puncture wounds prone to bacterial infection [18,19,39,40]; such injuries to the hand may progress to tenosynovitis, septic arthritis or osteomyelitis and not infrequently require hospitalisation or surgical debridement [41,42,43,44]; however, our registry did not systematically record wound infections, microbiological isolates, antibiotic use, or long-term outcomes, and these were therefore not assessed as outcomes of the present study, as in earlier work from this clinic [45].

Children differed from adults mainly in the pattern of injury localisation rather than in the overall use of PEP. The crude proportion receiving PEP did not differ between children and adults (35.9% versus 37.9%; χ^2^ = 0.56, *df* = 1, *p* = 0.46), whereas children were significantly more likely to sustain injuries to the head and neck (6.1% versus 1.3%; *p* < 0.001), which may reflect their smaller stature and closer face-to-animal contact during play [34,46]. Because head and neck exposures are categorised as more severe by the WHO and warrant prompt and complete PEP [5], this localisation pattern is the clinically relevant paediatric finding and supports individualised assessment in every child, even though the overall frequency of PEP was similar to that in adults.

The practical distinction between known-owner and unknown cats was the clearest factor associated with PEP use in our registry. Only 8.7% of patients injured by known-owner cats received PEP, compared with 62.0% after injuries caused by unknown cats. This difference reflects the central role of veterinary observation: when the animal can be identified, observed, or tested, unnecessary PEP can often be avoided, whereas when it cannot, clinicians understandably manage the exposure more cautiously. The same logic explains the 20 patients linked to a laboratory-confirmed rabid cat who did not receive PEP; as detailed in Section 3.3, these were contact-only registrations with no documented bite, scratch or mucosal exposure, and correctly did not meet WHO criteria for prophylaxis [5]. A comparable status-driven pattern, with an even higher overall PEP rate, was reported for bat exposures at the same clinic, consistent with the recommendation that every bat contact be treated as potentially rabid in the absence of laboratory exclusion [14].

The temporal trends coincide closely with the documented elimination of sylvatic rabies in Croatia. In jurisdictions where canine rabies has been controlled but wildlife and bat reservoirs persist, exposures to rabid or potentially rabid cats continue to account for a substantial share of human PEP and of animal rabies surveillance submissions [47,48,49]. In our series, all 150 patient records associated with laboratory-confirmed rabid cats were registered between 1995 and 2012, with a peak in 1997 (*n* = 26) and none thereafter, mirroring the decline of fox- and jackal-maintained rabies that followed the oral vaccination campaigns begun in 2011 [50], the last confirmed national animal rabies case in 2014, and the European Commission declaration of rabies-free status in 2021 [7,12,13]. HRIG use may be viewed as a more specific marker of high-concern exposures than vaccine use alone. Its decline to below 1% in 2015–2025, from 3.7% and 4.0% in the two earlier periods, closely tracks the disappearance of laboratory-confirmed rabid cats and suggests that severe rabies risk exposures became rare after elimination [5]. Vaccine-based PEP, by contrast, continued to be given to roughly one-third of patients. This persistence should not be interpreted as evidence of continuing endemic rabies, but rather as a likely reflection of uncertainty at the individual-exposure level, especially when the cat was stray, unavailable for observation, or of unknown origin. The proportion of patients receiving PEP nonetheless fell sharply in the final two years of the series (to approximately 13%); although too short to establish a trend, this recent decline may reflect evolving national post-exposure management practice [51] and warrants continued monitoring. Two features of the data help to explain this recent fall. First, the case-mix shifted toward lower-risk exposures: the share of presentations involving owned, observable cats rose from 45% in 2015–2023 to 63% in 2024–2025. Second, the propensity to administer PEP fell within every status category over the same comparison (from 68% to 30% for unknown cats and from 10% to 2% for owned cats), indicating a genuine lowering of the prophylaxis threshold rather than a change in case-mix alone. Both patterns are consistent with growing clinical confidence in withholding PEP for low-risk exposures once rabies-free status had consolidated, although the small number of patients in these two years (*n* = 146) means this remains a hypothesis to be confirmed by continued monitoring. Structured antirabies assessment remains relevant even in an officially rabies-free country. Although the risk of endemic rabies has been eliminated, uncertainty often persists at the level of the individual exposure, particularly when the offending cat is stray or unavailable for observation. Moreover, rabies-free status does not eliminate the possibility of virus reintroduction through cross-border movement of infected animals or, more rarely, spill-over of bat lyssaviruses into domestic cats reported elsewhere in Europe [15,22,23,52,53]. In Croatia, this framework is anchored in the Animal Health Act [54], which transposes Regulation (EU) 2016/429 and governs disease notification, surveillance, and the maintenance of disease-free status. These observations are a reminder that lyssavirus diversity and the global rabies burden remain a moving target even where classical RABV has been controlled [55,56]. Cat-scratch disease (*Bartonella henselae*), clinically relevant after cat exposures and documented in Croatian paediatric cohorts [57], illustrates that cat injuries carry infectious risks beyond rabies [58,59]; the corresponding registry variable was not analysed here because the consistency of its coding across the 31-year period could not be validated.

The temporal trends should not be interpreted in purely epidemiological terms, because several aspects of practice changed over the study period. The clinic operated under evolving national immunoprophylaxis programmes and WHO-aligned schedule revisions [51], oral vaccination of foxes was introduced in 2011 [50], the vaccine products in routine use changed, and the structure of the overall antirabies workload shifted, as reflected in the rising share of cat-related consultations despite falling absolute numbers (Section 3.1). Referral patterns, documentation practices and the catchment population are also likely to have changed. The observed changes in PEP and HRIG administration therefore reflect both genuine epidemiological change, above all the elimination of sylvatic rabies, and these secular changes in clinical protocols, referral and reporting, rather than epidemiological factors alone.

The main strengths of this study are its size (3147 patients over 31 uninterrupted years), its single-centre design, which limits between-institution variation in exposure assessment, and its origin at the national reference centre, where rabies exposure assessment follows established national and WHO-aligned practice. The study has several limitations. The retrospective, registry-based design restricts the analysis to routinely recorded variables; in particular, the registry does not separate bites from scratches, does not record injury severity, does not capture the WHO category I/II/III classification as a distinct variable, and does not include wound infections, microbiological data, antibiotic use, or long-term outcomes. Because the registry records only the doses administered at this clinic, the recorded dose count reflects clinic-administered doses rather than the completeness of the overall course, as patients may have begun or continued prophylaxis elsewhere, and although individual dose dates are recorded in the registry, they were not extracted for the present analysis, so dose timeliness was not assessed; the documented completion rate (69.9%) is therefore a lower bound. As a clinic-based registry it reflects persons who presented to the ARC and cannot provide population incidence, and the patterns may not generalise beyond the Zagreb region. In addition, because the series spans three decades of evolving clinical protocols, referral patterns and reporting practices, residual confounding of the temporal trends by these secular changes cannot be excluded; the multivariable model and the segmented time-series analysis describe the associations and the trend more formally but cannot fully separate epidemiological change from change in clinical practice. Finally, the registry did not record the vaccination status of the offending cats. Future integration of human-exposure records with veterinary data on cat ownership, observation and vaccination status would improve post-elimination rabies surveillance.

These findings also have practical implications for post-exposure prophylaxis in settings where rabies has been controlled. In the multivariable analysis the odds of PEP were about eighteen-fold higher for unknown than for owned cats, with only minor patient-level effects, so the main opportunity to avoid unnecessary prophylaxis lies in resolving the status of the animal through prompt owner identification and 10-day veterinary observation, rather than in patient-level triage. Unknown-cat exposures accounted for 896 of the 1183 PEP courses (76%), given at seven times the rate seen for owned cats (62.0% vs. 8.7%); this is the category in which resolving animal status offers the greatest scope to avoid prophylaxis, although how much is achievable in practice depends on the proportion of unknown cats that can actually be traced and observed, which these data cannot determine. In a rabies-free country the probability of true rabies exposure is very low, so reliable observation of owned cats can safely spare PEP, while genuinely unavailable animals remain the residual indication for it. The near-disappearance of HRIG after 2012 suggests that immunoglobulin, a costly and supply-limited product, can be reserved for the now-exceptional high-risk exposure. Because the Croatian setting, having moved from sylvatic endemicity through wildlife vaccination to rabies-free status, resembles that of many Central and Western European countries, these patterns are likely to apply to other regions in, or approaching, the post-elimination phase, where much of the continuing PEP is driven by unknown or unavailable animals rather than by demonstrable rabies risk. They are less applicable where dog-mediated rabies remains endemic or where bat-lyssavirus exposure predominates. In such settings, illustrated by the recent Colombian experience in which free-roaming cats bridged bat-maintained virus to fatal human cases [27], the priority shifts from exposure-driven prophylaxis toward vaccination of outdoor cats, so the management of cat injuries should be tailored to the local reservoir. More broadly, these three decades illustrate how the rationale for prophylaxis shifts as a country moves from endemic to rabies-free: PEP becomes increasingly driven by uncertainty about the animal rather than by demonstrable rabies risk, which is the central consideration for post-elimination programmes.

## 5. Conclusions

Cat-related injuries remained a stable component of antirabies practice across three decades, despite the elimination of sylvatic rabies in Croatia. Across this period, injured persons were predominantly women in mid-adulthood, most injuries involved the fist and fingers, and children were distinguished mainly by a higher proportion of head and neck injuries. The disappearance of laboratory-confirmed rabid cats after 2012 and the marked decline in HRIG use are consistent with the impact of successful rabies control, while the continued use of vaccine-based PEP reflects persistent uncertainty related mainly to unknown or unavailable cats. The clearest practical factor associated with PEP was the status of the offending cat, with PEP given to 8.7% of patients injured by known-owner cats compared with 62.0% after injuries by unknown cats, underlining the central role of veterinary observation in avoiding unnecessary prophylaxis. These findings support maintaining structured rabies exposure assessment and close coordination between human and veterinary surveillance, rather than routine escalation of all cat-related injuries, even within an officially rabies-free national context. In post-elimination settings, the most effective way to reduce unnecessary prophylaxis is to resolve the status of the animal through prompt owner identification and veterinary observation, rather than to escalate management for every cat-related injury.

## Figures and Tables

**Table 1:** Demographic and clinical characteristics of patients with cat-related injuries, Zagreb Antirabies Clinic, 1995–2025 (*N* = 3147).

Variable	*n*	%
**Sex**		
Female	2039	64.8
Male	1108	35.2
**Age group (years)**		
<18	462	14.7
18–34	730	23.2
35–49	661	21.0
50–64	747	23.7
≥65	547	17.4
**Animal status**		
Known owner	1525	48.5
Unknown	1444	45.9
Suspected of rabies	28	0.9
Laboratory-confirmed rabid	150	4.8
**Injury localisation**		
Fist and fingers	1872	59.5
Upper limbs	579	18.4
Lower limbs	419	13.3
Multiple sites	202	6.4
Head and neck	64	2.0
Trunk	11	0.3
**Clinical management**		
Post-exposure prophylaxis (PEP)	1183	37.6
Vaccine only	1091	34.7
Vaccine + HRIG	92	2.9
Human rabies immunoglobulin (HRIG)	92	2.9
Veterinary observation of animal	506	16.1
Animal tested for rabies	258	8.2
Positive	150	4.8
Negative	108	3.4
Hospitalisation	36	1.1

HRIG, human rabies immunoglobulin; PEP, post-exposure prophylaxis. Percentages are calculated from the total cohort (*N* = 3147). PEP comprised vaccine alone (*n* = 1091) or vaccine combined with HRIG (*n* = 92); all HRIG recipients also received vaccine. Animal-status categories refer to patient records (consultations), not to unique cats; a single rabid cat could be linked to several records.

**Table 2:** Patient characteristics and clinical management stratified by the status of the offending cat. *p*-values are from Pearson’s χ^2^ test, or Fisher’s exact test for comparisons with any expected cell count below five.

Variable	Known Owner (*n* = 1525)	Unknown (*n* = 1444)	Suspected (*n* = 28)	Confirmed Rabid (*n* = 150)	*p*-Value
Female sex, *n* (%)	1057 (69.3)	869 (60.2)	23 (82.1)	90 (60.0)	<0.001
Children <18, *n* (%)	199 (13.0)	217 (15.0)	6 (21.4)	40 (26.7)	<0.001
Fist/fingers, *n* (%)	834 (54.7)	926 (64.1)	14 (50.0)	98 (65.3)	<0.001
Head/neck, *n* (%)	42 (2.8)	21 (1.5)	0 (0)	1 (0.7)	0.057
PEP administered, *n* (%)	132 (8.7)	896 (62.0)	25 (89.3)	130 (86.7)	<0.001
HRIG administered, *n* (%)	4 (0.3)	9 (0.6)	12 (42.9)	67 (44.7)	<0.001
Veterinary observation, *n* (%)	436 (28.6)	70 (4.8)	0 (0)	0 (0)	<0.001
Hospitalisation, *n* (%)	20 (1.3)	15 (1.0)	1 (3.6)	0 (0)	0.22

PEP, post-exposure prophylaxis; HRIG, human rabies immunoglobulin. Categorical variables compared by Pearson’s χ^2^ test or Fisher’s exact test where appropriate. Among the 150 patients linked to a laboratory-confirmed rabid cat, 20 were contact-only registrations with no documented physical exposure and correctly did not receive PEP (see Section 3.3). Column *n* values denote patient records, not unique animals.

**Table 3:** Temporal trends in patient characteristics and clinical management across three decadal periods, 1995–2025.

Variable	1995–2004 (*n* = 1072)	2005–2014 (*n* = 1148)	2015–2025 (*n* = 927)	*p*-Value
Female sex, *n* (%)	682 (63.6)	744 (64.8)	613 (66.1)	0.50
Age (median, IQR)	43 (21–58)	44 (26–59)	44 (28–60)	0.005
Children <18, *n* (%)	224 (20.9)	153 (13.3)	85 (9.2)	<0.001
**Animal status**				**<0.001**
Known owner, *n* (%)	566 (52.8)	519 (45.2)	440 (47.5)	
Unknown, *n* (%)	414 (38.6)	549 (47.8)	481 (51.9)	
Suspected, *n* (%)	12 (1.1)	10 (0.9)	6 (0.6)	
Confirmed rabid, *n* (%)	80 (7.5)	70 (6.1)	0 (0)	
Fist/fingers, *n* (%)	638 (59.5)	720 (62.7)	514 (55.4)	0.004
Head/neck, *n* (%)	24 (2.2)	20 (1.7)	20 (2.2)	0.67
PEP administered, *n* (%)	328 (30.6)	508 (44.3)	347 (37.4)	<0.001
HRIG administered, *n* (%)	40 (3.7)	46 (4.0)	6 (0.6)	<0.001
Hospitalisation, *n* (%)	9 (0.8)	11 (1.0)	16 (1.7)	0.13

PEP, post-exposure prophylaxis; HRIG, human rabies immunoglobulin; IQR, interquartile range. Bold entries denote variable groupings. Categorical variables compared by Pearson’s χ^2^ test; age compared by Kruskal–Wallis test. The disappearance of laboratory-confirmed rabid cats after 2012 coincides with the documented elimination of sylvatic rabies in Croatia, culminating in the European Commission declaration of rabies-free status in 2021.

**Table 4:** Factors independently associated with post-exposure prophylaxis (PEP) administration: multivariable binomial logistic regression (*N* = 3147).

Variable	aOR (95% CI)	*p*-Value
**Animal status (ref. known owner)**		
Unknown	17.9 (14.4–22.2)	<0.001
Suspected	101.7 (29.9–345.3)	<0.001
Confirmed rabid	84.7 (50.2–143.0)	<0.001
Female sex (ref. male)	0.83 (0.68–1.00)	0.048
Age (per 10 years)	1.04 (1.00–1.09)	0.070
Injury site: multiple (ref. fist/fingers)	1.77 (1.21–2.58)	0.003
Injury site: head/neck (ref. fist/fingers)	0.50 (0.23–1.09)	0.082
Period 2005–2014 (ref. 1995–2004)	1.99 (1.60–2.49)	<0.001
Period 2015–2025 (ref. 1995–2004)	1.50 (1.19–1.90)	0.001

aOR, adjusted odds ratio; CI, confidence interval; ref., reference category. HRIG administration was not modelled multivariably because of the small number of events (*n* = 92) and its near-complete determination by animal status. The wide confidence interval for suspected cats reflects the small number of patients in that category (*n* = 28).

## Data Availability

The anonymised dataset analysed in this study contains sensitive registry-level information and is not publicly available, in compliance with applicable data-protection regulations. Aggregated data underlying the findings are available from the corresponding author upon reasonable request.
